# Safety and Efficacy of Two Ultrathin Biodegradable Polymer Sirolimus‐Eluting Stents in Real‐World Practice: Genoss DES Stents Versus Orsiro Stents From a Prospective Registry

**DOI:** 10.1002/clc.70060

**Published:** 2024-12-18

**Authors:** Ho Sung Jeon, Young Jin Youn, Jung‐Hee Lee, Young Jun Park, Jung‐Woo Son, Jun‐Won Lee, Min‐Soo Ahn, Sung Gyun Ahn, Jang‐Young Kim, Byung‐Su Yoo, Junghan Yoon

**Affiliations:** ^1^ Division of Cardiology, Department of Internal Medicine Yonsei University Wonju College of Medicine Wonju South Korea

**Keywords:** coronary artery disease, drug‐eluting stent, percutaneous coronary intervention, propensity score, prospective registries

## Abstract

**Background:**

The Orsiro and Genoss DES stents are biodegradable polymer drug‐eluting stents (DESs) with ultrathin struts.

**Objective:**

To investigate the safety and efficacy of these two ultrathin DESs in real‐world practice.

**Methods:**

From a single‐center prospective registry, we included 751 and 931 patients treated with the Genoss DES and Orsiro stents, respectively. After propensity score matching, we compared 483 patients in each group with respect to a device‐oriented composite outcome (DOCO), which comprised cardiac death, target vessel myocardial infarction, and clinically indicated target lesion revascularization up to 2 follow‐up years.

**Results:**

After propensity score matching, there were no significant between‐group differences in clinical and angiographic characteristics. During the median follow‐up period of 730 days (interquartile range, 427–730 days), there was no significant between‐group difference in the DOCO rate (3.1% in the Genoss DES group vs. 2.9% in the Orsiro group, log‐rank *p* = 0.847).

**Conclusions:**

This study demonstrated comparable safety and efficacy between the Orsiro and Genoss DES stents during a 2‐year follow‐up period in real‐world practice. However, this result should be confirmed in a large randomized controlled trial.

**Trial Registration:**

ClinicalTrials.gov Identifier: NCT02038127.

## Graphical abstract

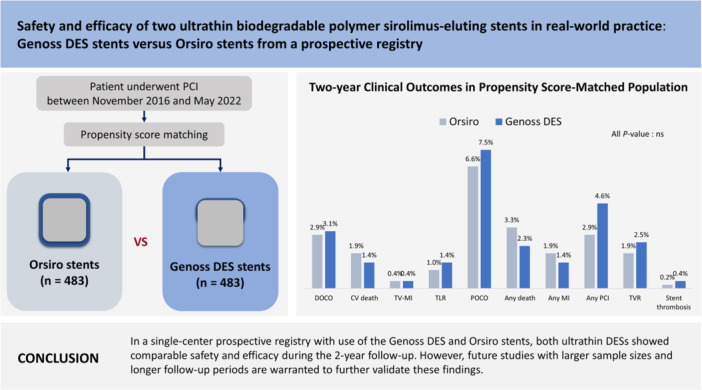

## 1 Introduction

Drug‐eluting stents (DES) are considered a standard treatment for patients undergoing percutaneous coronary intervention (PCI) [1]. Given the risk of very late stent thrombosis with DES due to delayed arterial healing related to hypersensitivity to durable polymers, biodegradable polymer DESs have been developed [2]. Although a biodegradable polymer DES with a thicker strut has an increased risk of stent thrombosis compared with durable polymer cobalt‐chromium everolimus‐eluting stents (EES), biodegradable polymer DES with an ultrathin strut involves a decreased rate of myocardial infarction (MI) and a trend towards reduced stent thrombosis compared with contemporary durable polymer DES [3, 4, 5].

The Orsiro stent (Biotronik AG, Bülach, Switzerland) is a biodegradable polymer DES with an ultrathin strut comprising a 60‐μm cobalt‐chromium L605 platform covered with an amorphous silicon carbide layer. It releases sirolimus from a biodegradable poly L‐lactic acid (PLLA) polymer. Several randomized controlled trials (RCTs) have shown that the Orsiro stent is superior or non‐inferior to the durable polymer EES and zotarolimus‐eluting stent (ZES) with respect to safety and efficacy in a broad spectrum of patients [6, 7, 8, 9, 10, 11, 12].

The Genoss DES stent (Genoss Company Limited, Suwon, South Korea) is another biodegradable polymer sirolimus‐eluting stent with an ultrathin strut. This biodegradable coating comprises a proprietary blend of poly (lactic‐co‐glycolic acid) and PLLA. Compared with the Orsiro stent, the Genoss DES stent has a slightly thicker cobalt‐chromium L605 platform (70 μm vs. 60 μm) but thinner polymer (3.0 μm vs. 7.4 μm). However, the polymer coatings of the Orsiro and Genoss DES stents are circumferential and abluminal, respectively. Accordingly, the Genoss DES stent has a lower total thickness (strut + polymer) than the Orsiro stent. In an interim analysis of a single‐arm prospective registry, the Genoss DES stent showed excellent safety and efficacy in all‐comer patients [13]. However, the Genoss DES stent was compared with the durable polymer platinum‐chromium EES (Promus Element stent, Boston Scientific, Natick, MA) in a small first‐in‐man trial [14].

This study aimed to compare the safety and efficacy of these two ultrathin DESs in real‐world practice.

## 2 Methods

### 2.1 Study Design and Population

From a prospective Gangwon PCI registry (ClinicalTrials.gov Identifier: NCT02038127), which enrolls patients undergoing PCI at three tertiary hospitals in Gangwon province, South Korea, we selected 4836 patients from a single center between November 2016 and May 2022. Among them, we selected 751 and 931 patients treated with the Genoss DES and Orsiro stents, respectively. The study protocol was approved by the local Institutional Review Board (CR320095) and conducted according to the principles of the Declaration of Helsinki revised in 2013. Written informed consent was obtained from all patients before enrollment in the registry.

### 2.2 Study Endpoints and Follow‐Up

The primary endpoint was a device‐oriented composite outcome (DOCO), which comprised cardiac death, MI not clearly attributable to a nontarget vessel, and clinically indicated target lesion revascularization. The secondary endpoints included a patient‐oriented composite outcome (POCO) comprising any death, MI, and revascularization; Academic Research Consortium‐defined stent thrombosis; and each component of the primary and secondary endpoints. Details and definitions of the endpoints have been described elsewhere [15].

Clinical follow‐ups were performed at 6, 12, and 24 months by office visits, medical record reviews, or telephone interviews. Data regarding the patients’ clinical status, interventions, and outcome events were recorded at every visit. Routine follow‐up angiography was not permitted.

### 2.3 PCI and Medical Treatment

PCI was performed according to the standard technique. Decisions regarding the vascular access site; pre‐dilatation or direct stenting; use of heparin or glycoprotein IIb/IIIa inhibitors; image guidance PCI; use of pressure wire, which is a technique for bifurcation or chronic total occlusion lesion; and stent selection were at the operator's discretion. A staged procedure was allowed and was not considered as revascularization. The selection and duration of antiplatelet therapy were at the physician's discretion. A loading dose of aspirin (300 mg) and P2Y12 inhibitor (clopidogrel [600 mg], prasugrel [60 mg], or ticagrelor [180 mg]) was administered to all patients ≤ 6 h before the procedure, unless the patient had been taking these medications or the procedure was an emergency situation. After the index PCI, dual antiplatelet therapy (DAPT) with a maintenance dose of aspirin (100 mg) and P2Y12 inhibitor (clopidogrel [75 mg QD], prasugrel [10 mg QD], or ticagrelor [90 mg BID]) was encouraged for ≥ 12 months. Moreover, guideline‐directed medical treatment with beta‐blockers, renin‐angiotensin‐aldosterone system inhibitors, and statins were encouraged.

### 2.4 Statistical Analyses

Categorical variables are presented as a number (percentages) and were compared using the chi‐squared test or Fisher's exact test. Continuous variables are presented as mean ± standard deviation or median (interquartile range) and were compared using the independent sample *t*‐test or Mann‐Whitney *U* test, as appropriate. Cumulative events of clinical outcomes were assessed using Kaplan–Meier estimates and compared using the log‐rank test. All analyses were truncated at 24 months of follow‐up owing to between‐group differences in the follow‐up duration. Analyses for all clinical endpoints were performed until the date of an endpoint event, loss to follow‐up, or up to 24 months after the index procedure, whichever came first. Propensity scores were estimated by fitting a logistic regression model with the clinical, angiographic, and procedural variables presented in Tables 1 and 2. Nearest‐neighbor matching with a caliper of 0.05 was used. To determine the balance in the established propensity score‐matched sample, standardized mean differences were used for between‐group comparisons of the means of the continuous and binary covariates. A standard difference of < 0.1 was considered indicative of negligible difference (Figure S1) [16]. Subgroup analyses of the primary endpoint were conducted based on age (< 65 years or ≥ 65 years), sex, diabetes mellitus, acute MI, stent diameter (> 2.5 mm vs. ≤ 2.5 mm), and length (< 38 mm vs. ≥ 38 mm). Statistical analyses were performed using SPSS, version 27.0 (IBM Corp., Armonk, N.Y., USA) and SAS, version 9.3 (SAS Institute Inc., Cary, NC, USA). A two‐sided *p* < 0.05 was considered statistically significant.

**TABLE 1:** Baseline clinical, angiographic, and procedural characteristics in propensity score‐matched population.

Variables	Orsiro *n* = 483	Genoss DES *n* = 483	*p*‐value
Male	343 (71.0)	350 (72.5)	0.617
Age, year	66.7 ± 11.5	66.7 ± 11.0	0.964
BMI, kg/m2	25.0 ± 3.4	25.0 ± 3.2	0.981
SBP, mmHg	142 ± 24	141 ± 24	0.688
DBP, mmHg	83 ± 14	83 ± 15	0.555
HR, BPM	77 ± 15	76 ± 15	0.232
Hypertension	284 (58.8)	272 (53.6)	0.435
Diabetes	165 (34.2)	159 (32.9)	0.683
Insulin‐dependent	12 (2.5)	16 (3.3)	0.443
Chronic kidney disease	92 (19.0)	99 (20.5)	0.572
Dialysis dependent	7 (1.4)	8 (1.7)	0.795
Dyslipidemia	161 (33.3)	157 (32.5)	0.784
Prior CVA	39 (8.1)	41 (8.5)	0.815
Current or ex‐smoker	305 (63.1)	313 (64.8)	0.592
Stable angina	69 (14.3)	70 (14.5)	0.927
Unstable angina	125 (25.9)	128 (26.5)	0.826
NSTEMI	151 (31.3)	141 (29.2)	0.484
STEMI	86 (17.8)	95 (19.7)	0.458
Primary PCI	71 (14.7)	79 (16.4)	0.477
Silent ischemia	33 (6.8)	33 (6.8)	> 0.999
CAG diagnosis			0.433
1‐VD	148 (30.6)	155 (32.1)	
2‐VD	157 (32.5)	169 (35.0)	
3‐VD	178 (36.9)	159 (32.9)	
Treated lesion			
LAD	328 (67.9)	313 (64.8)	0.307
LCX	132 (27.3)	133 (27.5)	0.943
RCA	175 (36.2)	175 (36.2)	> 0.999
LM	24 (5.0)	23 (4.8)	0.881
Moderate to severe angulation	7 (1.4)	4 (0.8)	0.363
Moderate to severe calcification	91 (18.8)	82 (17.0)	0.450
SYNTAX score	22.9 ± 14.8	21.9 ± 14.0	0.230
Multi‐vessel PCI	179 (37.1)	171 (35.4)	0.592
FFR guidance	49 (10.1)	18 (3.7)	< 0.001
IVUS guidance	298 (61.7)	267 (55.3)	0.043
CTO PCI	24 (5.0)	19 (3.9)	0.435
Bifurcation PCI	279 (57.8)	278 (57.6)	0.948
with 2‐stent strategy	11 (2.3)	12 (2.5)	0.833
Transradial access	470 (97.3)	463 (95.9)	
Implanted stent n	1.7 ± 0.9	1.7 ± 0.9	0.736
Implanted stent length, mm	46.6 ± 27.7	46.6 ± 30.0	0.990
Implanted stent diameter, mm	3.10 ± 0.39	3.09 ± 0.42	0.854
Periprocedural complication			
No reflow phenomenon	26 (5.4)	39 (8.1)	0.095
Side branch occlusion	17 (3.5)	21 (4.3)	0.508
Edge dissection	5 (1.0)	3 (0.6)	0.725
Perforation	3 (0.6)	3 (0.6)	> 0.999
Stent migration	5 (1.0)	0 (0)	0.062
Discharge medication			
Aspirin	459 (95.0)	465 (96.3)	0.344
Clopidogrel	266 (55.1)	258 (53.4)	0.605
Prasugrel or ticagrelor	209 (43.3)	221 (45.8)	0.437
Statin	440 (91.1)	437 (90.5)	0.739
Beta‐blocker	284 (58.8)	275 (56.9)	0.558
ACEi or ARB	290 (60.0)	294 (60.9)	0.792

*Note:* Values are n (%) and mean ± SD.

Abbreviations: ACEi, Angiotensin‐converting enzyme inhibitor; ARB, Angiotensin II receptor blocker; BMI, body mass index; CAG, coronary angiography; CTO, chronic total occlusion; CVA, cerebrovascular accident; DBP, diastolic blood pressure; DES, drug‐eluting stent; FFR, Fractional Flow Reserve; HR, heart rate; IVUS, intravascular ultrasound; LAD, left anterior descending artery; LCX, left circumflex artery; LM, left main; NSTEMI, Non‐ST‐segment elevation myocardial infarction; PCI, percutaneous coronary intervention; RCA, right coronary artery; SBP, systolic blood pressure; STEMI, ST‐segment elevation myocardial infarction; SYNTAX, SYNergy between PCI with TAXUS and Cardiac Surgery; VD, vessel disease.

**TABLE 2:** Clinical outcomes in propensity score‐matched population.

	Orsiro *n* = 483	Genoss DES *n* = 483	Log rank *p*‐value
Median follow‐up, days	730 (391, 730)	730 (497, 730)	
DAPT duration, days	370 (291, 519)	371 (321, 422)	0.503
In‐hospital death	0 (0)	0 (0)	n/a
Device‐oriented composite outcome^a^	14 (2.9)	15 (3.1)	0.847
Cardiovascular death	9 (1.9)	7 (1.4)	0.533
Target vessel‐related MI	2 (0.4)	2 (0.4)	0.921
Target lesion revascularization	5 (1.0)	7 (1.4)	0.697
Patient‐oriented composite outcome	32 (6.6)	36 (7.5)	0.989
Any death	16 (3.3)	11 (2.3)	0.262
Any MI	9 (1.9)	7 (1.4)	0.492
Any PCI	14 (2.9)	22 (4.6)	0.284
Target vessel revascularization	9 (1.9)	12 (2.5)	0.665
Stent thrombosis	1 (0.2)	2 (0.4)	0.597
Subacute definite	0 (0)	0 (0)	
Late definite	0 (0)	0 (0)	
Very late definite	0 (0)	1 (0.2)	
Subacute probable	1 (0.2)	0 (0)	

*Note:* Values are *n* (%) or median (interquartile range).

Abbreviations: DAPT, dual antiplatelet therapy; DES, drug‐eluting stent; MI, myocardial infarction; PCI, percutaneous coronary intervention.

a Primary endpoint.

## 3 Results

**FIGURE 1:**
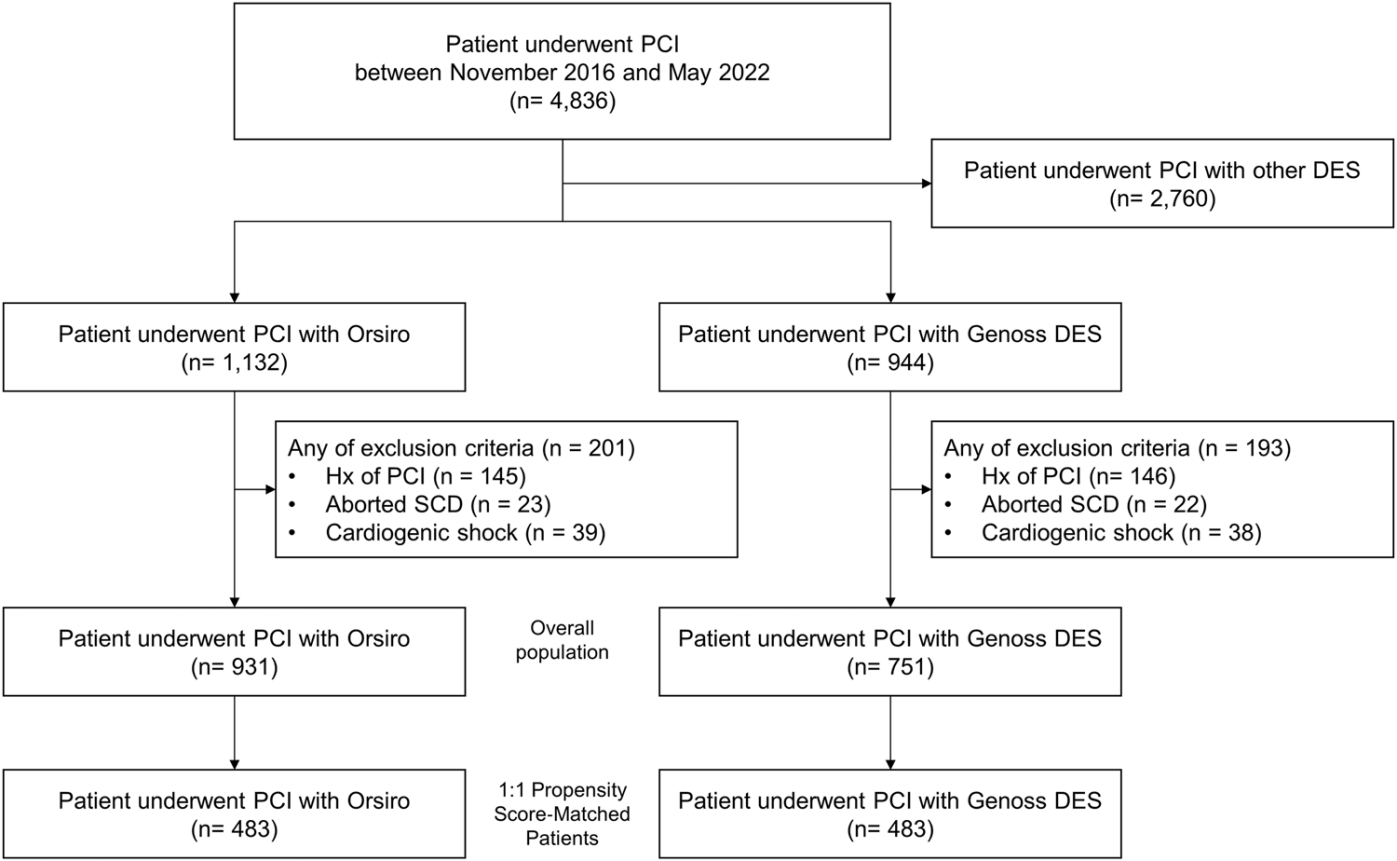
Study flow diagram. A total of 1682 patients were assessed for the primary endpoint. After 1:1 propensity score matching using the clinical variables, 483 patients in each group were compared. DES, drug‐eluting stent; PCI, percutaneous coronary intervention; SCD, sudden cardiac death.

As shown in Figure 1, 931 and 751 patients in the Orsiro and Genoss DES groups, respectively, were compared as a crude population after excluding the patients with a prior history of PCI, aborted sudden cardiac death, or cardiogenic shock to reduce potential bias. Finally, 483 patients in either group were compared after propensity score matching.

### 3.1 Overall Population

Baseline clinical, angiographic, and procedural characteristics in a crude population are presented in Supporting Information S1: Table 1. The mean age was 66.7 ± 11.4 years, with 71.1% of the patients being male. Approximately 57.4% of the patients had hypertension, 34.5% had diabetes, 20.6% had chronic kidney disease, and 62.2% were either current or ex‐smokers.

Non‐ST‐segment elevation acute coronary syndrome, multi‐vessel disease, and multi‐vessel PCI were more prevalent in the Orsiro group than in the Genoss DES group. Accordingly, the implanted stent number and length were significantly higher in the Orsiro group than in the Genoss DES group. The left anterior descending artery was the most commonly treated lesion in both groups, with a higher prevalence in the Orsiro group than in the Genoss DES group. However, left main artery disease was more prevalent in the Genoss DES group than in the Orsiro group. The rate of moderate‐to‐severe angulation of the treated lesions was similar between the two groups. However, the rate of moderate‐to‐severe calcification was more prevalent in the Orsiro group than in the Genoss DES group. Fractional flow reserve (FFR) and intravascular ultrasound (IVUS) guidance for PCI were more prevalent in the Orsiro group than in the Genoss DES group. However, the occurrence of periprocedural complications, including the no‐reflow phenomenon, side branch occlusion, edge dissection, perforation, and stent migration, was not different between the two groups.

Medication usage at discharge was different between the two groups (Supporting Information S1: Table 2). The Genoss DES group had a higher prescription rate of clopidogrel, while the Orsiro group had a higher prescription rate of potent P2Y12 inhibitors (Prasugrel or ticagrelor). Additionally, statins and beta‐blockers were prescribed more frequently in the Orsiro group than in the Genoss DES group.

**FIGURE 2:**
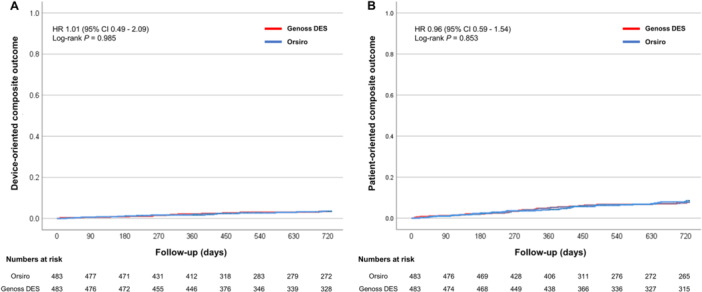
Kaplan–Meier curves of adverse clinical events for propensity score‐matched populations. Event rates of (A) device‐oriented composite outcome consisting of cardiac death, myocardial infarction not clearly attributable to a nontarget vessel, and clinically indicated target lesion revascularization; and (B) patient‐oriented composite outcome consisting of any death, any myocardial infarction, and any revascularization in patients implanted with the Genoss DES (red lines) and Orsiro (blue lines), during the 2‐year follow‐up. CI, confidence interval; DES, drug‐eluting stent; HR, hazard ratio.

Despite the between‐group differences in baseline clinical, angiographic, and procedural characteristics, there were no between‐group differences in the DOCO rate (4.3% vs. 5.1%; hazard ratio [HR], 0.88; 95% confidence interval [CI], 0.57–1.38; log‐rank *p* = 0.581) and POCO rate (6.5% vs. 8.7%; HR, 1.01; 95% CI, 0.73–1.40; log‐rank *p* = 0.964) over a median follow‐up period of 730 days (interquartile range, 427–730 days) (Supporting Information S1: Table 3 and Supporting Information S1: Figure 2). Other clinical outcomes did not significantly differ between the two groups.

### 3.2 Propensity Score‐Matched Population

After propensity score matching, the two groups were well matched in baseline clinical, angiographic, and procedural characteristics, except for a lower rate of physiology‐ and image‐guided PCI in the Genoss DES group than in the Orsiro group. Furthermore, medication usage at discharge was well matched (Table 1).

**FIGURE 3:**
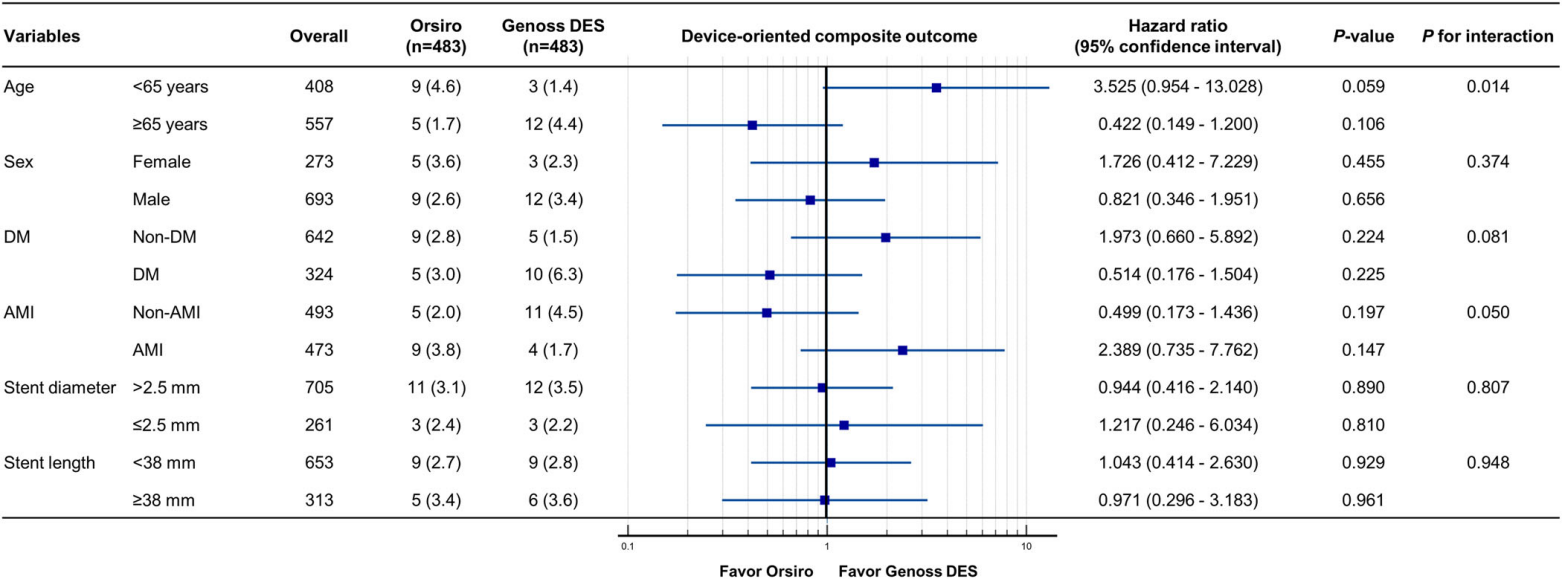
Subgroup analysis for device‐oriented composite outcomes at 2 years. Data are presented as number of events (% of patients). A device‐oriented composite outcome consisting of cardiovascular death, myocardial infarction, and target lesion revascularization was not clearly attributable to a nontarget lesion. AMI, acute myocardial infarction; DES, drug‐eluting stent; DM, diabetes mellitus.

Clinical outcomes are presented in Table 2 and Figure 2. During a median follow‐up period of 730 days (interquartile range, 427–730 days), the DOCO rate (2.9% vs. 3.1%, HR 1.01; 95% CI, 0.49–2.09; log‐rank *p* = 0.985) and POCO rate (6.6% vs. 7.5%, HR 0.96; 95% CI, 0.59–1.54; log‐rank, *p* = 0.853) were similar between the two groups, with a similar duration of DAPT. Furthermore, other clinical outcomes were similar between the two groups. The rates of DOCO were consistent across the subgroups, except for those based on age (Figure 3).

## 4 Discussion

This study presents the first comparison between the ultrathin Genoss DES and ultrathin Orsiro stents in real‐world practice over an extended follow‐up period. The main finding was that the Genoss DES stent had similar safety and efficacy profiles at 24 follow‐up months compared with the Orsiro stent after propensity score matching.

The Orsiro stent has been compared with the standard of care—a thin strut of either the durable polymer EES (Xience Prime/Xpedition stent, Abbott, Abbott Park, IL, USA) or ZES (Resolute Integrity/Onyx stent, Medtronic, Santa Rosa, CA, USA)—in several large RCTs [6, 7, 8, 9, 10, 11, 12]. In the first large, international, randomized non‐inferiority BIOFLOW V trial, the Orsiro stent showed a significantly lower rate of target lesion failure (TLF) than the Xience stent (6.2% vs. 9.6%, *p* = 0.04), which was mainly attributed to the between‐stent difference in target vessel MI (5% vs. 8%) at 12 months [9]. This finding was consistent at the 2‐year follow‐up of the BIOFLOW V trial [17]. However, these two stents showed a similar rate of TLF in the all‐comer BIOSCIENCE trial (6.5% vs. 6.8%) [6]. In a subgroup analysis of the BIOSCIENCE trial, the Orsiro stent showed a lower rate of TLF than the Xience stent in 407 patients with ST‐segment elevation MI (STEMI) (3.4% vs. 8.8%) [18]. The subsequent BIOSTEMI trial compared these two stents in patients with STEMI undergoing primary PCI [11]. Bayesian analysis of the STEMI subgroup in the BIOSCIENCE trial confirmed that the Orsiro stent was superior to the Xience stent in terms of TLF (4% vs. 6%). The BIONYX trial compared the Orsiro stent with the novel single‐wire designed ZES (Resolute Onyx stent) in an all‐comer population [10]. Although increased definite or probable stent thrombosis was observed with the Orsiro stent (0.7% vs. 0.1%), both stents showed similar target vessel failure rates (4.7% vs. 4.5%, respectively) at 12 months. The three‐arm BIO‐RESORT trial compared the Orsiro stent with another biodegradable polymer platinum‐chromium thinner strut (74 μm**)** EES (Synergy stent, Boston Scientific, Natick, MA, USA) and the Resolute Integrity stent [8]. At the 3 follow‐up years, the Orsiro stent showed the lowest rate of TLF (7.0%, Orsiro group: 9.5%, Synergy group: 10.0%, Resolute Integrity group) [19].

The favorable outcomes of the Orsiro stent might be mainly attributed to its thinner strut and biodegradable polymer, regardless of the difference in drug and stent design. Although the Genoss DES stent has a slightly thicker strut than the Orsiro stent, the polymer is thinner and coated on the abluminal stent; accordingly, it has a lower total stent thickness. Therefore, considering the stent thickness, a favorable clinical outcome could be expected. In a small first‐in‐man trial, angiographic and clinical outcomes at 9 months were similar between the Genoss DES (*n* = 38) and Promus Element stents (*n* = 39) [14]. At 5 follow‐up years, these two stents showed a similar rate of adverse clinical outcomes (5.3% in the Genoss DES stent vs. 12.8% in the Promus Element stent, *p* = 0.431) [20]. Given the small sample size, a prospective multicenter single arm Genoss DES registry study was subsequently conducted in South Korea. In an interim analysis of this registry, the Genoss DES stent showed excellent clinical outcomes (0.6% of DOCO, 3.9% of POCO, and 0.6% of definite or probable stent thrombosis) in 622 all‐comer patients at 1 follow‐up year [13]. In the final analysis of this registry, the Genoss DES stent showed consistently excellent clinical outcomes (1.8% for DOCO, 4.0% for POCO, and 0.4% for definite or probable stent thrombosis) among 1999 all‐comer patients at the 1‐year follow‐up [21]. Given the lack of a comparator in the Genoss DES registry, the present study was conducted. Our findings demonstrated similar safety and efficacy of the Genoss and Orsiro stents in a real‐world practice up to the 2‐year follow‐up period.

This study has several limitations. First, despite utilizing propensity score matching in our patient selection from the prospective registry, the potential for selection bias remains. Notably, the use of FFR and IVUS guidance was higher in the Orsiro group, likely reflecting operator preference. However, it is important to note that neither FFR nor IVUS guidance were significant predictors of the DOCO in this study. In addition, our subgroup analysis revealed an interaction for age in the DOCO (< 65 years vs. ≥ 65 years), which was not evident in the overall population. Second, the overall event rates observed were relatively low, even though this was a prospective registry trial based on real‐world practice. This could be attributed to several reasons: (1) the exclusion of procedure‐related MI from our analysis; (2) the use of potent ADP‐receptor antagonists in approximately 50% of the patients; (3) the predominant use of radial access (over 90%); and (4) possible ethnic or genetic factors that may be associated with lower clinical event rates. These elements should be considered when interpreting the results of our study.

## 5 Conclusion

In a single‐center prospective registry with use of the Genoss DES and Orsiro stents, both ultrathin DESs showed comparable safety and efficacy during the 2‐year follow‐up period. However, future studies with larger sample sizes and longer follow‐up periods are warranted to further validate these findings.

## Conflicts of Interest

The authors declare no conflicts of interest.

## Supporting information

Supporting information.

Supporting information.

Supporting information.

Supporting information.

## Data Availability

The data that support the findings of this study are available from the corresponding author upon reasonable request.
