# Supplementary material for: Safety and Efficacy of Two Ultrathin Biodegradable Polymer Sirolimus‐Eluting Stents in Real‐World Practice: Genoss DES Stents Versus Orsiro Stents From a Prospective Registry
Source: Clin Cardiol. 2024 Dec 18;47(12):e70060. doi: 10.1002/clc.70060 (PMC11652947; doi:10.1002/clc.70060)
Supplement: Supplementary file 1 — Supporting information. [file CLC-47-e70060-s003.docx]

**Supplementary Table 1. Baseline clinical, angiographic, and procedural characteristics** **in overall population**

| Variables | Orsiro n = 931 | Genoss DES n = 751 | *p*-value |
| --- | --- | --- | --- |
| Male | 666 (71.5) | 530 (70.6) | 0.665 |
| Age, year | 66.4 ± 11.5 | 67.1 ± 11.1 | 0.155 |
| BMI, kg/m2 | 24.9 ± 3.4 | 25.0 ± 3.3 | 0.302 |
| SBP, mmHg | 141 ± 24 | 141 ± 24 | 0.642 |
| DPB, mmHg | 83 ± 14 | 83 ± 15 | 0.657 |
| HR, BPM | 78 ± 16 | 77 ± 16 | 0.580 |
| Hypertension | 538 (57.8) | 427 (56.9) | 0.701 |
| Diabetes | 323 (34.7) | 258 (34.4) | 0.884 |
| Insulin dependent | 24 (2.6) | 24 (3.2) | 0.449 |
| Chronic kidney disease | 186 (20.5) | 154 (20.7) | 0.906 |
| Dialysis dependent | 25 (2.7) | 13 (1.7) | 0.190 |
| Dyslipidemia | 327 (35.1) | 251 (33.4) | 0.465 |
| Prior CVA | 84 (9.0) | 66 (8.8) | 0.867 |
| Current or ex-smoker | 570 (61.2) | 477 (63.5) | 0.431 |
| Stable angina | 112 (12.0) | 107 (14.2) | 0.179 |
| Unstable angina | 244 (26.2) | 244 (32.5) | 0.005 |
| NSTEMI | 318 (34.2) | 202 (26.9) | 0.001 |
| STEMI | 169 (18.2) | 124 (16.5) | 0.378 |
| Primary PCI | 152 (16.3) | 100 (13.3) | 0.085 |
| Silent ischemia | 53 (5.7) | 49 (6.5) | 0.477 |
| CAG diagnosis |  |  | 0.047 |
| 1-VD | 266 (28.6) | 253 (27.2) |  |
| 2-VD | 302 (32.4) | 248 (26.6) |  |
| 3-VD | 363 (39.0) | 250 (26.9) |  |
| Treated lesion |  |  |  |
| LAD | 654 (70.2) | 481 (51.7) | 0.007 |
| LCX | 275 (29.5) | 200 (21.5) | 0.188 |
| RCA | 354 (38.0) | 256 (27.5) | 0.095 |
| LM | 39 (4.2) | 48 (5.2) | 0.043 |
| Moderate to severe angulation | 14 (1.5) | 6 (0.6) | 0.185 |
| Moderate to severe calcification | 193 (20.7) | 125 (13.4) | 0.033 |
| Multi-vessel PCI | 391 (42.0) | 249 (26.7) | <0.001 |
| FFR guidance | 64 (6.9) | 26 (2.8) | 0.002 |
| IVUS guidance | 605 (65.0) | 392 (42.1) | <0.001 |
| CTO PCI | 48 (5.2) | 34 (3.7) | 0.552 |
| Bifurcation PCI | 569 (61.1) | 435 (46.7) | 0.184 |
| with 2-stent strategy | 14 (1.5) | 40 (4.3) | <0.001 |
| Transradial access | 706 (75.8) | 709 (76.2) | 0.531 |
| Implanted stent n | 1.8 ± 1.0 | 1.7 ± 1.0 | 0.005 |
| Implanted stent length, mm | 52.9 ± 32.2 | 44.3 ± 29.1 | <0.001 |
| Implanted stent diameter, mm | 3.06 ± 0.39 | 3.10 ± 0.42 | 0.023 |
| Periprocedural complication |  |  |  |
| No reflow phenomenon | 56 (6.0) | 45 (6.0) | 0.984 |
| Side branch occlusion | 40 (4.3) | 27 (3.6) | 0.465 |
| Edge dissection | 12 (1.3) | 10 (1.3) | 0.939 |
| Perforation | 4 (0.4) | 4 (0.5) | >0.999 |
| Stent migration | 5 (0.2) | 2 (0.3) | 0.471 |

Values are n (%) and mean ± SD.

BMI, body mass index; CAG, coronary angiography; CTO, chronic total occlusion; CVA, cerebrovascular accident; DBP, diastolic blood pressure; DES, drug-eluting stent; FFR, Fractional Flow Reserve; HR, heart rate; IVUS, intravascular ultrasound; LAD, left anterior descending; LCX, left circumflex; LM, left main; NSTEMI, Non-ST-segment elevation myocardial infarction; PCI, percutaneous coronary intervention; RCA, right coronary artery; SBP, systolic blood pressure; STEMI, ST-segment elevation myocardial infarction; VD, vessel disease**.**

**Supplementary Table 2.** **Discharge medication among survivors in overall population**

| Variables | Orsiro n = 931 | Genoss DES n = 751 | *p*-value |
| --- | --- | --- | --- |
| Aspirin | 876 (95.2) | 710 (95.5) | 0.475 |
| Clopidogrel | 423 (46.0) | 431 (58.2) | <0.001 |
| Prasugrel or ticagrelor | 479 (52.1) | 300 (40.5) | <0.001 |
| Statin | 864 (93.9) | 561 (78.8) | <0.001 |
| Beta-blocker | 589 (64.0) | 396 (53.5) | <0.001 |
| ACEi or ARB | 561 (61.0) | 447 (60.4) | 0.812 |

Values are n (%).

ACEi, Angiotensin-converting enzyme inhibitor; ARB, Angiotensin II receptor blocker; DES, drug-eluting stent.

**Supplementary Table 3. Clinical outcomes** **in overall population**

|  | Orsiro n = 931 | Genoss DES n = 751 | Log rank  *p*-value |
| --- | --- | --- | --- |
| Median follow-up, days | 730 (406, 730) | 730 (516, 730) |  |
| DAPT duration, days | 370 (291, 519) | 375 (331, 421) | 0.974 |
| In-hospital death | 11 (1.2) | 11 (1.5) | 0.611 |
| Device-oriented composite outcome* | 40 (4.3) | 38 (5.1) | 0.576 |
| Cardiovascular death | 29 (3.1) | 23 (3.1) | 0.811 |
| Target vessel-related MI | 4 (0.4) | 2 (0.3) | 0.543 |
| Target lesion revascularization | 12 (1.3) | 14 (1.9) | 0.450 |
| Patient-oriented composite outcome | 78 (6.5) | 65 (8.7) | 0.971 |
| Any death | 49 (5.3) | 34 (4.5) | 0.419 |
| Any MI | 17 (1.8) | 8 (1.1) | 0.150 |
| Any PCI | 27 (2.9) | 28 (3.7) | 0.475 |
| Target vessel revascularization | 17 (1.8) | 19 (2.5) | 0.436 |
| Stent thrombosis | 1 (0.1) | 5 (0.7) | 0.059 |
| Subacute definite | 0 (0) | 1 (0.1) |  |
| Late definite | 0 (0) | 0 (0) |  |
| Very late definite | 0 (0) | 1 (0.1) |  |
| Subacute probable | 1 (0.1) | 2 (0.3) |  |

Values are n (%) or median (interquartile range).

*Primary endpoint.

DAPT, dual antiplatelet therapy; DES, drug-eluting stent; MI, myocardial infarction; PCI, percutaneous coronary intervention.
