# Supplementary figures and images for: Safety and Efficacy of Two Ultrathin Biodegradable Polymer Sirolimus‐Eluting Stents in Real‐World Practice: Genoss DES Stents Versus Orsiro Stents From a Prospective Registry
Source: Clin Cardiol. 2024 Dec 18;47(12):e70060. doi: 10.1002/clc.70060 (PMC11652947; doi:10.1002/clc.70060)

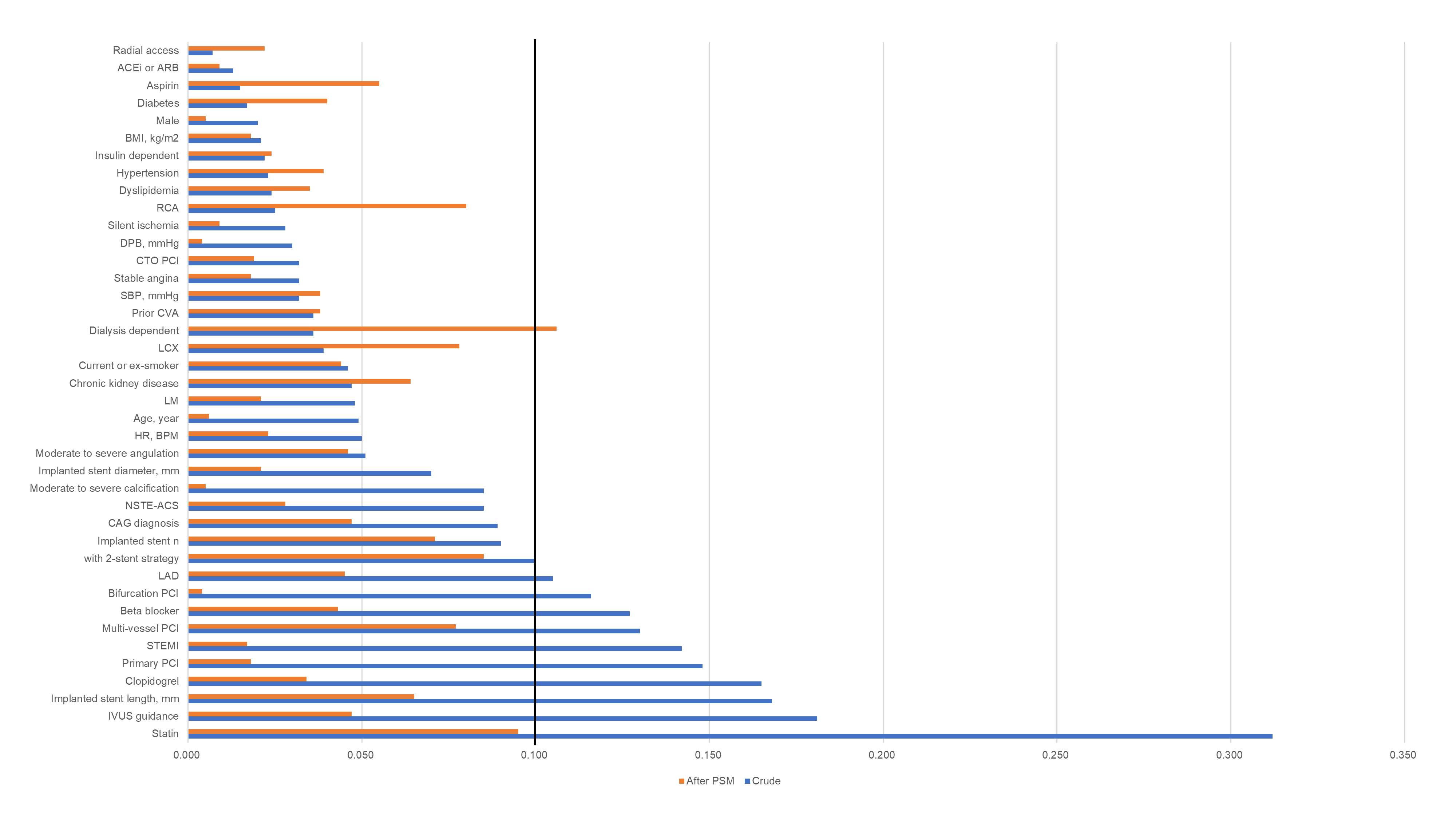

Supplement: Supplementary file 2 — Supporting information. [file CLC-47-e70060-s001.TIF]

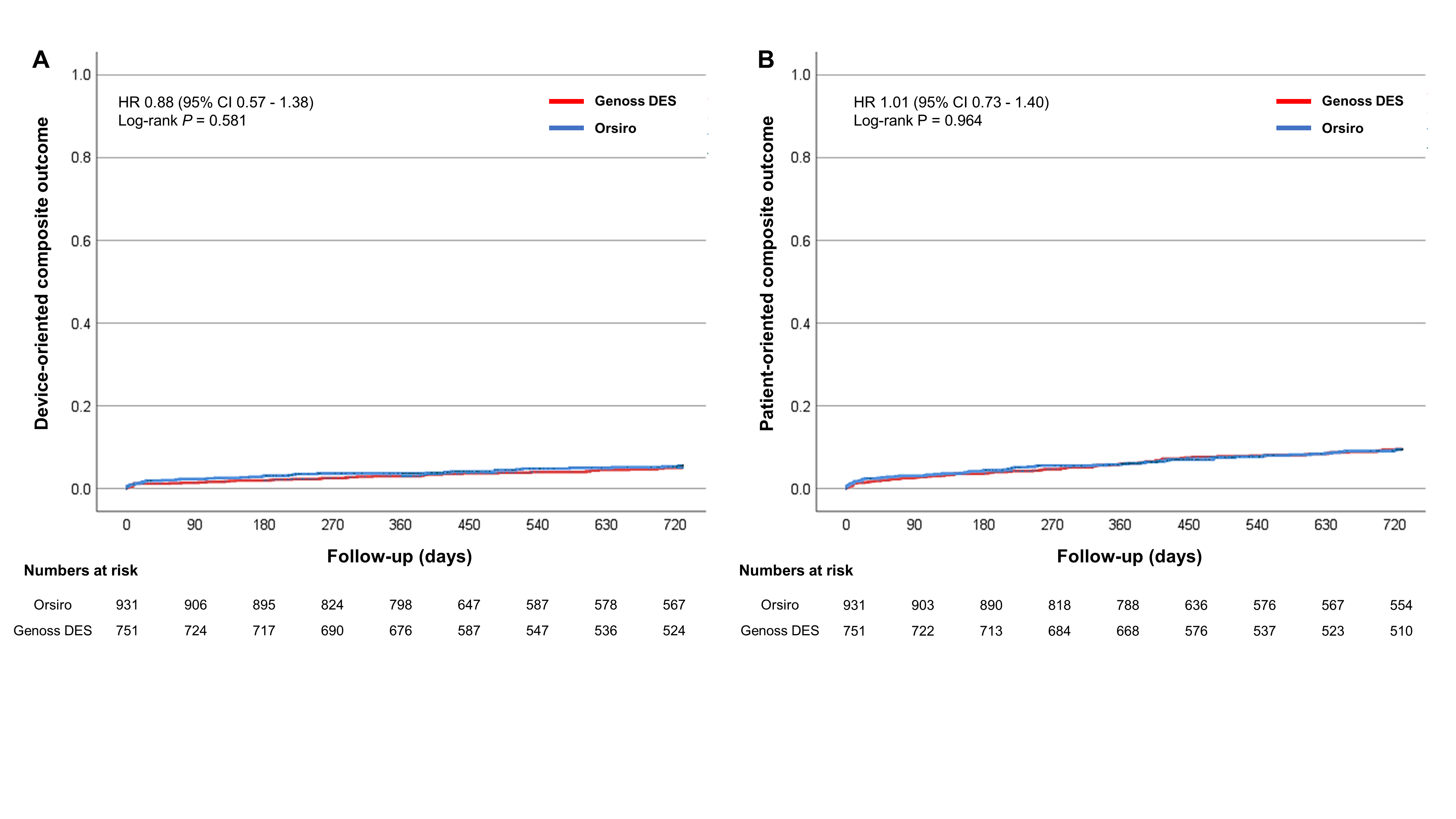

Supplement: Supplementary file 3 — Supporting information. [file CLC-47-e70060-s004.TIF]
