# Supplementary material for: Safety and Efficacy of Two Ultrathin Biodegradable Polymer Sirolimus‐Eluting Stents in Real‐World Practice: Genoss DES Stents Versus Orsiro Stents From a Prospective Registry
Source: Clin Cardiol. 2024 Dec 18;47(12):e70060. doi: 10.1002/clc.70060 (PMC11652947; doi:10.1002/clc.70060)
Supplement: Supplementary file 4 — Supporting information. [file CLC-47-e70060-s002.docx]

**Supplementary Figure Legends**

**Supplementary Figure 1. Standard difference before and after propensity score-matching**

ACEi, angiotensin-converting enzyme inhibitor; ARB, angiotensin II receptor blocker; BMI, body mass index; CAG, coronary angiography; CTO, chronic total occlusion; CVA, cerebrovascular accident; DBP, diastolic blood pressure; HR, heart rate; IVUS, intravascular ultrasound; LAD, left anterior descending; LCX, left circumflex; LM, left main; NSTE-ACS, Non-ST-segment elevation acute coronary syndrome; PCI, percutaneous coronary intervention; RCA, right coronary artery; SBP, systolic blood pressure; STEMI, ST-segment elevation myocardial infarction..

**Supplementary Figure 2. Kaplan–Meier curves of adverse clinical events for propensity score-matched populations.**

Event rates of (A) device-oriented composite outcome consisting of cardiac death, myocardial infarction not clearly attributable to a non-target vessel, and clinically indicated target lesion revascularization; and (B) patient-oriented composite outcome consisting of any death, any myocardial infarction, and any revascularization in patients implanted with the Genoss DES (red lines) and Orsiro (blue lines), during the 2-year follow-up.

DES, drug-eluting stent
